# Improved Point Cloud Representation via a Learnable Sort–Mix–Attend Mechanism

**DOI:** 10.3390/s26061888

**Published:** 2026-03-17

**Authors:** Yuyan Zhang, Xi Wang, Zhang Yi, Lei Xu

**Affiliations:** 1 Machine Intelligence Laboratory, College of Computer Science, Sichuan University, Chengdu 610065, China; 2 Department of Computer Science and Engineering, The Hong Kong University of Science and Technology, Hong Kong, China

**Keywords:** point clouds, deep learning, canonicalization, local aggregation, representation learning, 3D segmentation, 3D object classification, scene understanding

## Abstract

Recent years have seen remarkable progress in deep learning on 3D point clouds, with hierarchical architectures becoming standard. Most work has focused on developing increasingly complex operators, such as self-attention, while enhancing the representational capacity of efficient point-wise MLP-based backbones has received less attention. We address this issue by proposing a differentiable module that learns to impose a task-driven canonical structure on local point sets. Our proposed SMA (Sort–Mix–Attend) layer dynamically serializes a neighborhood by generating a geometric basis and using a differentiable sorting mechanism. This enables an efficient MLP-based network to model rich feature interactions, adaptively modulating features prior to the final symmetric aggregation function. We demonstrate that SMA effectively enhances standard backbones for 3D classification and segmentation. Specifically, integrating SMA into PointNeXt-S achieves an Overall Accuracy (OA) of 88.3% on the challenging ScanObjectNN dataset, an improvement of 0.6% over the baseline. Furthermore, it boosts the classic PointNet++ architecture by a significant 5.2% in OA. We also introduce a highly efficient SMA-Tiny variant that achieves 86.0% OA with only 0.3 M parameters, proving the structural superiority, computational cost-effectiveness, and practical significance of our method for real-world 3D perception tasks.

## 1. Introduction

The proliferation of advanced 3D sensing technologies, such as LiDAR and RGB-D cameras, has made raw point clouds a primary data modality for the intelligent processing, sensing, and understanding of complex physical environments [1,2]. Efficiently and accurately analyzing this unstructured sensor data is crucial for robust perception in real-world applications, ranging from autonomous driving to robotic navigation and remote sensing [3]. However, the massive scale of raw point clouds presents foundational challenges—such as sparsity, inherent noise, incomplete geometry, and the critical necessity for data compression to manage large-scale storage and transmission [4,5]. Furthermore, comprehensive surveys have recently detailed advancements in point cloud enhancement [6], denoising [7], registration [8], and data augmentation [9]. In this context, hierarchical networks, epitomized by PointNet++ [10], have become a foundational architecture for 3D analysis due to their computational efficiency. Their core set abstraction (SA) layer learns local geometric patterns by applying a shared Multi-Layer Perceptron (MLP) to individual points, followed by max-pooling. However, point-wise MLPs process each point in isolation—a property we term context-agnostic feature learning. Consequently, the subsequent max-pooling layer aggregates unrefined, non-collaborative features, fundamentally limiting the network’s expressive power [11].

To capture richer inter-point dependencies and overcome the scalability bottlenecks of early architectures, the field has evolved significantly towards more complex interaction operators. Graph-based networks [12] and curve-driven models like CurveNet [13] successfully modeled explicit local geometric relations, while specialized continuous convolutions [14] and sparse voxel-based convolutions, epitomized by MinkowskiNet [15], provided robust structural processing. To further scale local feature aggregation to massive, real-world point clouds, architectures such as RandLA-Net [16] and ASSANet [17] introduced highly efficient random sampling and anisotropic separable set abstractions. Concurrently, to capture long-range dependencies, Transformer-based architectures [18,19,20,21,22,23] employed self-attention to capture global context. Innovations aiming to reduce the immense quadratic computational burden of these attention models have rapidly emerged. These include Parameter-Efficient Fine-Tuning (PEFT) methods like PointLoRA [24], and ultra-low-energy Spiking Point Transformers [25].

Concurrently, sequence-based paradigms, particularly State Space Models (SSMs), have gained immense traction for processing point sets with linear complexity [26,27,28,29]. To adapt these 1D sequence models for robust 3D structural modeling, researchers have recently proposed multi-scale shifted windows (WinMamba) [30] and pre-serialization Voxel Mamba [31]. However, transforming an unordered point set into a sequence in these models still heavily relies on fixed, predefined heuristics, such as space-filling Hilbert [32] or Morton curves [33]. While mathematically consistent, such data-agnostic ordering is fundamentally disconnected from the task-specific learning objective.

To provide a clear perspective on the current landscape, Table 1 summarizes representative advancements, explicitly outlining the trade-offs between interaction complexity, architectural efficiency, and serialization strategies.

Problem Statement and Objectives: As highlighted in our review, there remains a critical gap between the computational efficiency of context-agnostic MLP models and the heavy computational burden (or rigid heuristic constraints) of complex interaction models. The primary goal of this study is to bridge this gap by introducing a rich, context-aware interaction mechanism into MLP-based architectures in a fully end-to-end learnable manner. The specific objectives of this research are threefold: (1) to develop a differentiable, data-driven serialization module that replaces fixed mathematical heuristics; (2) to design a lightweight mixer that leverages this canonical sequence for structural feature refinement; and (3) to validate the theoretical superiority and practical efficiency of this mechanism over established baselines across multiple 3D perception tasks. By addressing these objectives, we aim to transform the standard max-pooling operation from a simple maximum filter into a structurally-informed selection mechanism.

The remainder of this article is organized as follows: Section 2 details the study materials, experimental setup, and the phenomenological methodology of the proposed SMA architecture. Section 3 presents the objective quantitative results obtained across multiple benchmarking datasets. Section 4 systematically discusses these results, emphasizing the fundamental theoretical novelty and comparative advantages of our approach. Finally, Section 5 summarizes our core conclusions and outlines prospects for future research.

## 2. Materials and Methods

**Figure 1:**
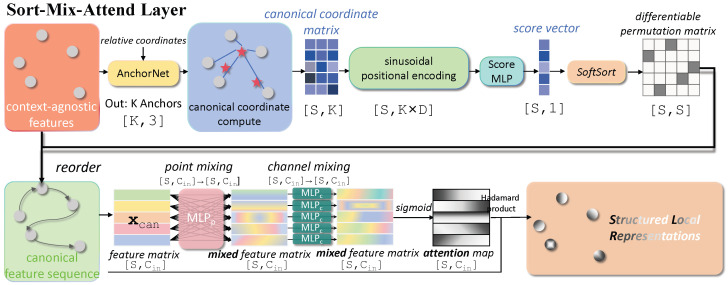
The overall architecture of the SMA layer. The top row illustrates the differentiable canonicalization module, which takes relative coordinates to produce a differentiable permutation matrix. The bottom row shows the Interaction and Refinement stage, where the initial features are reordered into a canonical sequence, processed by an MLP-based mixer to capture structural context, and then used to generate a refined feature map for the final aggregation. The red stars denote the dynamically generated spatial anchor points. Different background colors are used to visually distinguish distinct functional modules.

To address the limitations of existing 3D point cloud architectures, our research program follows a systematic phenomenological and methodological approach. First, we phenomenologically analyze the feature extraction bottleneck in standard PointNet-like models (detailed in Section 2.2). Based on this mathematical observation, we meticulously design the Sort–Mix–Attend (SMA) layer—a differentiable canonicalization and interaction module (detailed in Section 2.3). Finally, we conduct rigorously controlled empirical experiments across varying scales of spatial environments (objects, parts, and indoor scenes) to validate the theoretical superiority of our proposed architecture. The macro-level algorithmic workflow of the SMA layer is systematically illustrated in Figure 1 and step-by-step in Listing 1.

**Listing 1.** PyTorch-style pseudo-code of the SMA layer forward pass.

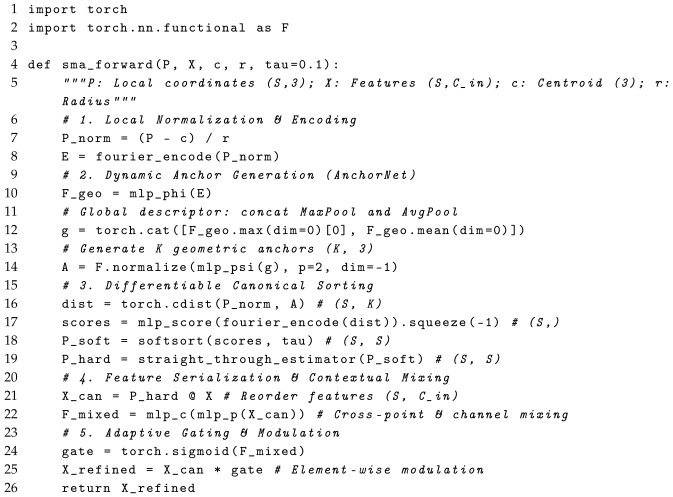



### 2.1. Study Materials and Experimental Setup

To rigorously evaluate the proposed Sort–Mix–Attend (SMA) architecture, we selected four standard benchmarking datasets that provide a comprehensive spectrum of geometric complexity and real-world applicability. This deliberate selection justifies the model’s effectiveness across diverse engineering scenarios:

- **ModelNet40** [36]: Serving as our baseline material, this dataset comprises 12,311 synthetically generated, clean CAD models across 40 categories. It is utilized to validate the fundamental geometric feature extraction capabilities of the model in an idealized, noise-free environment.
- **ScanObjectNN** [37]: As our primary testbed for real-world robustness, this dataset contains 2902 point clouds derived from actual indoor scans. We utilize the most challenging variant (PB_T50_RS), which includes background noise, occlusions, and diverse spatial transformations. This material is critical for proving the practical viability of our method in non-ideal sensing scenarios.
- **ShapeNetPart and S3DIS**: The ShapeNetPart dataset [38] (16,881 shapes, 50 part labels) and the Stanford Large-Scale 3D Indoor Spaces (S3DIS) dataset [39] were selected to extend our evaluation beyond object-level classification to fine-grained part and scene-level dense segmentation, respectively.

All phenomenological models and algorithmic enhancements were implemented using the PyTorch 1.10 deep learning framework. The experimental setup consisted of a workstation equipped with dual NVIDIA RTX 3090 GPUs. To strictly isolate the theoretical and empirical contribution of the SMA layer, all base models enhanced with our module (+SMA) were trained using identical hyperparameter configurations and preprocessing steps as their respective original baselines.

### 2.2. Background and Motivation: Rethinking Local Aggregation

Processing unstructured 3D point clouds presents unique challenges due to their permutation-invariant nature and irregular spatial distribution. PointNet [40], a seminal work, addressed permutation invariance by applying point-wise Multi-Layer Perceptrons (MLPs) independently to each point, followed by a symmetric max-pooling operation. While computationally efficient, this approach inherently neglects crucial local geometric context, as point features are extracted in isolation.

PointNet++ [10] built upon PointNet by introducing hierarchical set abstraction (SA) modules to incorporate local contextual information. In a typical SA layer, a set of centroids $\left\{c_{j}\right\}$ is first sampled from the input points. Then, for each centroid $c_{j}$, a local neighborhood $N_{j}$ is defined by grouping all points within a ball of radius *r*:

(1) $$N_{j}=\{\left(p_{i},f_{i}\right)|\;∥p_{i}-c_{j}{∥}_{2}\;\leq \;r\},$$

where $p_{i}\in R^{3}$ are the point coordinates and $f_{i}\in R^{C_{\mathrm{in}}}$ are their associated feature vectors. Let $S=\;|N_{j}|$ be the number of points in the neighborhood. The local aggregation process is then expressed as:

(2) $$f_{\mathrm{agg},j}=\rho \left({\left\{h_{\Theta }\left(f_{i},p_{i}-c_{j}\right)\right\}}_{i\in N_{j}}\right),$$

where $h_{\Theta }:R^{C_{\mathrm{in}}}\times R^{3}\to R^{C_{\mathrm{out}}}$ is a learnable point-wise transformation (typically an MLP) with parameters Θ, and ρ is a symmetric reduction function (e.g., max-pooling).

This formulation has a critical limitation stemming from the independent application of $h_{\Theta }$ to each point. The feature transformation is context-agnostic, meaning that the transformed feature for a point $\left(p_{i},f_{i}\right)$ is conditionally independent of all other points in the neighborhood $N_{j}\backslash \left\{\left(p_{i},f_{i}\right)\right\}$, given its own attributes. This independence prevents the network from modeling local geometric or feature-space interactions until the final pooling operation ρ, which acts as an information bottleneck and may discard critical structural cues. Our SMA layer addresses this by introducing a structured, learnable representation of the unordered point set, enabling rich, context-aware feature interactions before the final aggregation.

### 2.3. The SMA Layer

The SMA layer addresses the aforementioned limitation by introducing a structured, learnable representation of the unordered point set, enabling rich, context-aware feature interactions before the final aggregation. As illustrated in Figure 1, it consists of input normalization and encoding, a differentiable canonicalization module, and a context-aware interaction and refinement stage.

#### 2.3.1. Input Normalization and Encoding

To ensure robustness to scale and to capture high-frequency geometric details, we first normalize and encode the input coordinates. Given a neighborhood centroid $c_{j}$ and a grouping radius r>0, the normalized relative coordinates for a point $p_{i}$ are:

(3) $${\mathrm{dp}}_{i,\mathrm{norm}}=\frac{p_{i}-c_{j}}{r}.$$

To mitigate the spectral bias of MLPs against high-frequency signals [41], we apply a sinusoidal positional encoding $\gamma :R^{3}\to R^{D}$ to these coordinates. The encoding function is defined as:

(4) $$\gamma \left(v\right)=\left[\sin(2\pi (v\cdot B)),\cos(2\pi (v\cdot B))\right],$$

where $B\in R^{3\times m}$ is a fixed random Fourier basis matrix with m=D/2, and its entries are sampled from a normal distribution $N(0,\sigma^{2})$. The output $e_{i}=\gamma \left({\mathrm{dp}}_{i,\mathrm{norm}}\right)\in R^{D}$ is a high-dimensional vector that enables MLPs to better capture high-frequency components in the data.

#### 2.3.2. Differentiable Canonicalization Module

We achieve canonicalization through two stages: dynamic anchor learning and differentiable sorting. Canonicalization transforms an unordered set of points $N_{j}$ into a deterministically ordered sequence based on a learned, data-driven geometric basis.

##### Dynamic Anchor Learning

**Figure 2:**
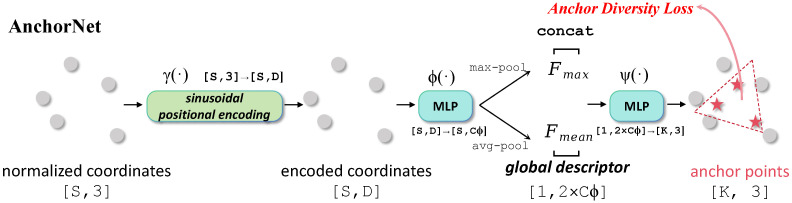
Architecture of the AnchorNet. It takes normalized coordinates, encodes them to overcome spectral bias, and then uses a permutation-invariant structure (point-wise MLP followed by concatenated max and average pooling) to generate a global descriptor. This descriptor is then used to regress the final anchor points, which are regularized by a diversity loss. The red stars denote the generated spatial anchor points.

An *AnchorNet*, detailed in Figure 2, generates a set of *K* ordered anchor points $A={\left\{a_{k}\right\}}_{k=1}^{K}$ that form a geometric basis for the neighborhood. The process is as follows:

AnchorNet first processes the encoded positions ${\left\{e_{i}\right\}}_{i=1}^{S}$ with an MLP $\phi :R^{D}\to R^{C_{\phi }}$ to extract point-wise geometric features. These are aggregated into a global descriptor $g\in R^{2C_{\phi }}$ that captures both salient and average properties of the point set’s geometry:

(5) $$g=\left[\max_{i=1}^{S}\left\{\phi \left(e_{i}\right)\right\}∥{\mathrm{avg}}_{i=1}^{S}\left\{\phi \left(e_{i}\right)\right\}\right],$$

where || denotes concatenation. A second MLP, $\psi :R^{2C_{\phi }}\to R^{K\times 3}$, regresses the raw anchor positions from the global descriptor: $A_{raw}=\psi \left(g\right)$. The raw anchors are projected into the unit ball using a norm-clipping projection operator $\Pi :R^{3}\to R^{3}$:

(6) $$a_{k}=\Pi \left(a_{raw,k}\right)=\frac{a_{raw,k}}{\max(1,∥a_{raw,k}{∥}_{2})}.$$

To prevent the learned anchors from collapsing into a degenerate state (i.e., $a_{i}\approx a_{j}$ for i≠j), we introduce an **Anchor Diversity Loss**, $L_{diversity}$, which encourages spatial separation:

(7) $$L_{diversity}=\sum_{i=1}^{K}\sum_{j=i+1}^{K}\exp\left(-\frac{∥a_{i}-a_{j}{∥}_{2}^{2}}{\sigma_{\mathrm{div}}^{2}}\right),$$

where $\sigma_{\mathrm{div}}$ is a hyperparameter defining the radius of repulsion.

##### Differentiable Sorting

Using the non-degenerate anchor set A, we compute a canonical coordinate matrix $C\in R^{S\times K}$, where each entry $C_{ik}={∥{\mathrm{dp}}_{i,\mathrm{norm}}-a_{k}∥}_{2}$ is the Euclidean distance from the normalized point *i* to anchor *k*.

These distances are encoded using γ(·) and then mapped to a scalar score vector $s\in R^{S}$ by a *Scoring* MLP, $M_{\mathrm{score}}$:

(8) $$s_{i}=M_{\mathrm{score}}\left(\left[\gamma \left(C_{i1}\right)∥\ldots ∥\gamma \left(C_{iK}\right)\right]\right).$$

The scalar scores are converted into a differentiable permutation matrix $\hat{P}\in R^{S\times S}$ using softsort [42], a continuous relaxation of the argsort operation:

(9) $$\hat{P}=\mathrm{softmax}\left(\frac{-d(\mathrm{sort}\left(s\right)1^{⊤},1s^{⊤})}{\tau }\right),$$

where d(·, ·) is a distance matrix and τ is a hyperparameter controlling the temperature.

##### Properties of the Module

The design of the canonicalization module ensures two critical properties. First, the entire process, from anchor generation to the final soft permutation matrix, is fully differentiable. This allows the parameters of both the AnchorNet and the Scoring MLP to be optimized end-to-end via gradient descent, enabling the network to learn a task-specific ordering. Second, the module as a whole is permutation-invariant. Since the AnchorNet generates a consistent set of anchors regardless of the input point order, the subsequent relational scoring and sorting process will always produce a permutation matrix that maps any input permutation of a given point set to the same canonical sequence.

#### 2.3.3. Context-Aware Interaction and Refinement

The pseudo-code of the SMA layer forward pass is summarized in Listing 1. With the input features $X\in R^{S\times C_{\mathrm{in}}}$ reordered into a canonical sequence $X_{\mathrm{can}}=\hat{P}X$, we can now model inter-point dependencies. To achieve this efficiently, we employ an MLP-based interaction network. This network is composed of two distinct MLP blocks. The first block, a point-mixing MLP (${\mathrm{MLP}}_{p}$), is applied across the spatial dimension of *S* points for each channel independently to facilitate inter-point communication. The second block, a channel-mixing MLP (${\mathrm{MLP}}_{c}$), is then applied to each point’s feature vector independently to model inter-channel correlations. Stacking these blocks produces a contextually rich representation

(10) $$F_{\mathrm{mixed}}={\mathrm{MLP}}_{c}\left({\mathrm{MLP}}_{p}\left(X_{\mathrm{can}}\right)\right).$$

This contextual information is then used to refine the features. $F_{\mathrm{mixed}}$ is passed through a sigmoid function to produce an attention map $A_{g}\in {\left[0,1\right]}^{S\times C_{\mathrm{in}}}$. This map adaptively modulates the canonically sorted features via element-wise multiplication (Hadamard product ⊙):

(11) $$X_{\mathrm{refined}}=X_{\mathrm{can}}⊙A_{g}.$$

This gated modulation transforms the subsequent max-pooling function from a simple maximum filter into a structurally-aware selection mechanism.

#### 2.3.4. Implementation Details

In our model, we set the number of anchor points to K=4. For Anchor Diversity Loss, we set $\sigma_{\mathrm{div}}=0.25$. For the differentiable sorting, we use a temperature of τ=0.1 in softsort. To obtain a discrete permutation matrix for the forward pass while preserving differentiability, we apply a Straight-Through Estimator (STE) [43] to the softsort output. This produces a doubly stochastic permutation matrix during inference while using the soft matrix for gradient computation during training.

The interaction network is constructed as follows. The point-mixing MLP, ${\mathrm{MLP}}_{p}$, consists of two linear layers of size S×S with a ReLU activation in between. The channel-mixing MLP, ${\mathrm{MLP}}_{c}$, uses a bottleneck design with a channel reduction factor of 8. Specifically, it comprises a linear layer that reduces the channel dimension from $C_{\mathrm{in}}$ to $C_{\mathrm{in}}/8$, followed by a ReLU activation, and another linear layer that projects it back to $C_{\mathrm{in}}$.

## 3. Results

To comprehensively evaluate the proposed SMA layer, we present quantitative performance metrics alongside qualitative visual analyses. This dual approach ensures a transparent assessment of the model’s empirical efficiency and its structural representation capabilities.

### 3.1. Object Classification

Table 2 presents the comparative classification results on both the ScanObjectNN (PB_T50_RS) and ModelNet40 datasets. Integrating the SMA layer into standard backbones yielded consistent performance gains, particularly on the noisy and occluded ScanObjectNN dataset. Most notably, the PointNeXt-S (+SMA) model achieved an Overall Accuracy (OA) of 88.3% and a mean class accuracy (mAcc) of 86.5%, representing a 0.6% OA improvement over its baseline. Furthermore, the integration boosted the legacy PointNet++ architecture by a significant 5.2% in OA, demonstrating the layer’s capacity to upgrade early context-agnostic models. Our custom ultra-lightweight variant, SMA-Tiny, achieved an impressive 86.0% OA utilizing only 0.3 M parameters and 0.6 G FLOPs.

### 3.2. Dense Scene and Part Segmentation

The fine-grained segmentation performance is quantitatively detailed in Table 3 for ShapeNetPart and Table 4 for S3DIS Area-5. On the ShapeNetPart dataset, the SMA-enhanced PointNet++ demonstrated a 0.6% increase in instance mIoU and a 1.2% increase in class mIoU. The modern PointNeXt-S (+SMA) reached an instance mIoU of 86.7%. For complex indoor scene parsing on S3DIS, PointNeXt-S (+SMA) achieved a 0.8% improvement in mIoU, reaching 63.4%. Consistent performance gains were also observed across the larger PointNeXt-B and PointNeXt-L variants.

### 3.3. Computational Efficiency Analysis

**Figure 3:**
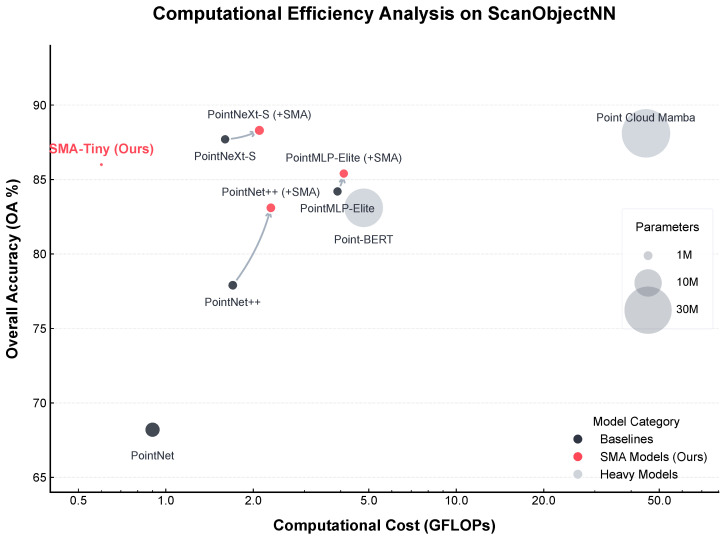
Performance versus parameter count on ScanObjectNN. Models enhanced with the SMA layer achieve higher parameter efficiency compared to complex mechanisms. The arrows indicate the performance improvement trajectory from the baseline models to their corresponding SMA-enhanced versions.

To visually contextualize the trade-off between model accuracy and computational cost, Figure 3 plots the Overall Accuracy of various models against their parameter counts on the ScanObjectNN dataset. Models enhanced with the SMA layer achieve higher parameter efficiency compared to complex attention mechanisms. This explicitly demonstrates that the SMA mechanism achieves superior or highly competitive structural efficiency without the massive parameter overhead characteristic of recent Transformer or strict attention-based models.

### 3.4. Ablation Studies

To systematically validate the key architectural design choices of the SMA layer, we conducted a series of in-depth ablation studies on the ScanObjectNN dataset, utilizing PointNeXt-S as the baseline model.

**Efficacy of Learnable Serialization vs. Heuristics.** A core claim of our methodology is that a learned, task-driven ordering is superior to fixed mathematical heuristics. We evaluated this by replacing our learnable AnchorNet serialization module with predefined space-filling curves, keeping the rest of the SMA architecture fixed. As shown in Table 5, the full SMA model with its differentiable sorting significantly outperforms alternative heuristics, yielding an OA of 88.3%. This objective data confirms that dynamic, data-driven serialization is critical for optimal structural representation.

**Analysis of the Gating Function.** We further investigated the design of the feature refinement gating function ($A_{g}$). An intuitive hypothesis is that a gating function with a range of [0, 2], such as 1+tanh(z) or 2×Sigmoid(z), might allow the network to simultaneously suppress unimportant features and amplify prominent ones. However, the empirical results presented in Table 6 demonstrate that the standard Sigmoid function with a strictly suppressive range of [0, 1] is the most effective. Removing the gate entirely (Identity) drops the performance to 87.5%.

**Figure 4:**
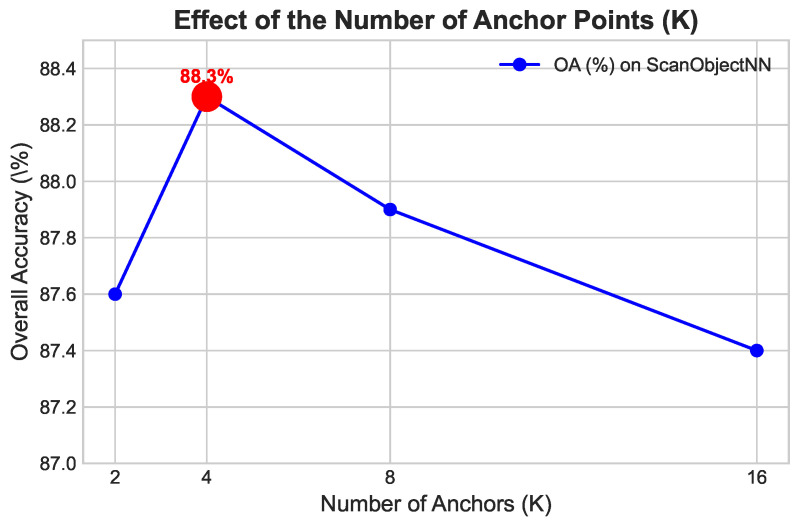
Ablation study on the number of anchor points (*K*). Performance peaks at K=4, defining the optimal balance between the expressive power of the geometric basis and the model’s optimization complexity.

**Effect of the Number of Anchor Points** (*K*). The number of anchor points (*K*) determines the complexity of the learned geometric basis. We varied *K* to objectively measure its impact on model performance. As illustrated in Figure 4, performance steadily improves as *K* increases from 2 to 4, indicating that a moderately complex basis effectively captures local structural nuances. However, increasing *K* further to 8 and 16 results in slight performance degradation, suggesting that an overly complex basis introduces redundancy or complicates the optimization landscape of the scoring function. Consequently, K=4 was established as the optimal structural balance.

### 3.5. Qualitative Visualizations

To complement the quantitative tables and provide intuitive insights into the operational improvements driven by the SMA layer, we present a series of qualitative visual comparisons.

**Figure 5:**
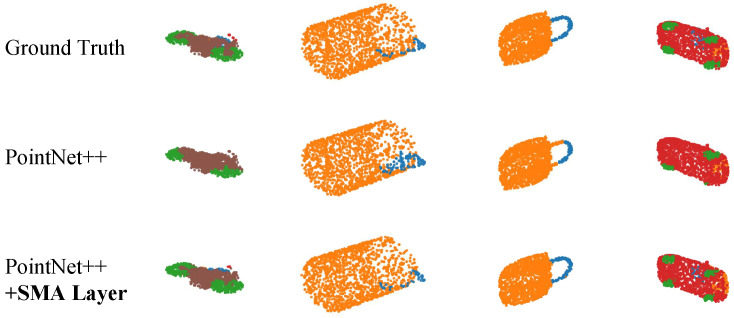
Qualitative comparison of part segmentation on ShapeNetPart. From left to right: ground truth (GT), baseline PointNet++, and PointNet++ enhanced with SMA. The SMA layer demonstrates finer boundary delineation and reduces misclassifications in complex local regions. Different colors represent different predicted part categories.

**Part Segmentation (ShapeNetPart):** Figure 5 illustrates the part segmentation results comparing the ground truth (GT), the baseline PointNet++ model, and the PointNet++ (+SMA) model. The visual evidence indicates that the baseline model frequently struggles with boundary delineation between adjacent parts and exhibits scattered misclassifications in geometrically complex regions. In contrast, the integration of the SMA layer results in smoother, more accurate part boundaries that closely align with the ground truth, highlighting its enhanced ability to capture fine-grained local geometric context.

**Figure 6:**
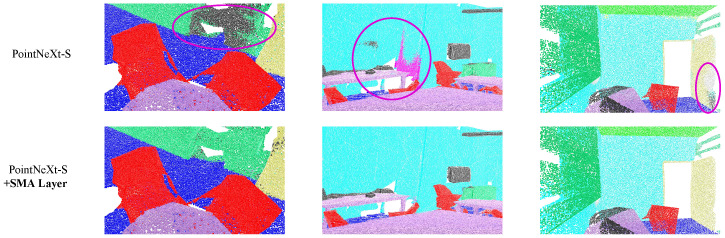
Qualitative comparison of semantic segmentation results on S3DIS. Purple circles highlight challenging regions where the SMA-enhanced model corrects baseline misclassifications. Different colors represent different predicted semantic categories.

**Semantic Segmentation (S3DIS):** Figure 6 presents a qualitative comparison of semantic segmentation in complex indoor environments. Challenging regions with intricate geometric structures and multi-category boundaries (highlighted by purple circles) demonstrate noticeably more accurate segmentation after integrating SMA.

**Figure 7:**
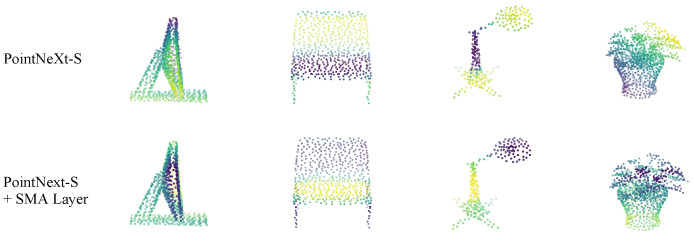
Feature activation visualization. The color mapping represents learned feature activations (darker colors indicate higher activations). The SMA layer significantly focuses activations on structurally discriminative regions.

**Feature Activation:** Finally, to visualize the internal feature representations, Figure 7 plots the feature activations of both the trained baseline PointNeXt-S and PointNeXt-S with SMA. The baseline model exhibits gradual, diffuse activation patterns that do not clearly distinguish structural elements. Conversely, the SMA layer helps focus the activations, concentrating higher activation values densely on the discriminative parts of the object necessary for accurate classification.

## 4. Discussion

The comprehensive quantitative metrics and qualitative visualizations obtained across multiple 3D perception benchmarks demonstrate the effectiveness of the SMA layer. By contextualizing our findings within the current literature (Table 1), several key insights regarding point cloud representation learning emerge.

### 4.1. Overcoming the Bottleneck of Context-Agnostic Pooling

Traditional hierarchical models, such as PointNet++ [10], rely on point-wise MLPs followed by symmetric max-pooling. This formulation treats the pooling operation as a static, “blind” filter that merely extracts the highest numerical activation, ignoring the collaborative geometric structure of the local neighborhood. Our results suggest that the historical bottleneck in early 3D point cloud processing was not necessarily a lack of network depth, but rather this isolated, non-collaborative nature of feature extraction prior to pooling.

The 5.2% OA jump observed when integrating SMA into the classic PointNet++ architecture supports this conclusion. As evidenced by the feature activation map (Figure 7), passing canonically sorted features through our MLP-mixer generates an attention gate that explicitly suppresses structurally irrelevant outlier points. This transforms local aggregation into a structurally informed selection mechanism, concentrating the network’s focus on discriminative geometric boundaries before the pooling bottleneck occurs.

### 4.2. The Necessity of Task-Driven Serialization

While SMA successfully resolves the aforementioned pooling bottleneck, its core enabler is the dynamic serialization process. Recent architectures, particularly State Space Models (SSMs), have popularized the conversion of 3D point clouds into 1D sequences. However, these models typically force point clouds into fixed, data-agnostic mathematical heuristics, such as Hilbert [32] or Morton Z-order curves [33]. Our core proposition is that spatial proximity does not strictly equate to structural importance.

By introducing the differentiable softsort process guided by a dynamically generated AnchorNet, the SMA layer imposes a canonical, task-driven sequence on the unordered set. This geometric basis is explicitly optimized via the backpropagated loss of the specific perception task. This theoretical stance is empirically proven by our ablation studies: our learnable anchor-based sorting yielded 88.3% OA on ScanObjectNN, notably outperforming the rigid Morton Z-order (87.3%) and Hilbert curves (87.8%). This confirms that data-driven serialization captures complex geometric nuances far better than predefined spatial heuristics.

### 4.3. Robustness to Spatial Unevenness and Compression

Beyond improving baseline accuracy, the dynamic serialization introduced in SMA provides critical resilience against data degradation. As extensively analyzed in recent comprehensive surveys and scalable LiDAR point cloud compression (LPCC) frameworks [4,5], raw LiDAR data is characterized by extreme sparsity and spatial unevenness. Compression algorithms (e.g., octree-based quantization or autoencoder downsampling) inevitably exacerbate this uneven distribution by merging adjacent points and altering local densities to reduce storage.

Such severe spatial unevenness exposes a critical vulnerability in the aforementioned fixed space-filling curves. Because these heuristics partition 3D space using rigid, uniform grids, compression-induced density shifts can drastically disrupt the structural continuity of the mapped 1D sequence. In contrast, the SMA module is inherently adaptive. Since the AnchorNet operates on normalized relative coordinates and aggregates localized descriptors using symmetric functions—which are mathematically robust to density variations and missing points—it guarantees a stable, context-aware ordering even under compression. This relative sorting mechanism makes our architecture highly resilient to the artifacts commonly introduced by modern LPCC pipelines.

### 4.4. Balancing Expressivity and Computational Efficiency

Finally, in addition to algorithmic robustness, the practical value of the SMA layer is underscored by its parameter efficiency, disrupting the current field-wide trend of exponentially scaling up parameter counts. As visualized in the computational efficiency analysis (Figure 3), recent Transformer-based models (e.g., Point-BERT [20]) and massive SSMs (e.g., Point Cloud Mamba [27]) rely on tens of millions of parameters and heavy pre-training paradigms to model complex global interactions.

Conversely, our custom SMA-Tiny architecture achieved a highly competitive 86.0% OA utilizing merely 0.3 Million parameters. The SMA mechanism achieves the highly expressive interaction capabilities typical of complex attention mechanisms but retains the ultra-low memory and latency footprint of pure MLPs. This makes our theoretically grounded approach exceptionally suitable for real-time engineering applications on resource-constrained edge devices, such as autonomous driving systems and robotic navigation.

## 5. Conclusions

In this study, we proposed the Sort–Mix–Attend (SMA) layer to fundamentally overcome the context-agnostic limitations of traditional MLP-based 3D point cloud architectures. By introducing a differentiable, dynamically generated serialization module, we successfully transformed the standard local aggregation process. Our research yields the following key conclusions:

1. We developed an end-to-end differentiable serialization module that dynamically generates a task-driven geometric sequence via an AnchorNet. This theoretically and empirically bypasses the structural limitations of fixed mathematical sorting heuristics currently prevalent in sequence-based point cloud models.
2. Integrating the SMA layer acts as a universal performance multiplier. It improved the classic PointNet++ by an impressive 5.2% Overall Accuracy on the challenging ScanObjectNN dataset, and pushed the modern PointNeXt-S to a state-of-the-art 88.3% OA. Consistent gains in structural boundary delineation were also observed in dense part (ShapeNetPart) and semantic (S3DIS) segmentation tasks.
3. We demonstrated that modeling complex geometric interactions does not necessitate massive parameter counts. Our custom SMA-Tiny network achieved 86.0% OA utilizing merely 0.3 Million parameters and 0.6G FLOPs, offering a highly cost-effective and practical solution for structural feature extraction compared to heavy Transformer-based attention models.
4. Despite its empirical success, the SMA layer faces inherent scalability constraints. The differentiable canonicalization, specifically the generation of a dense S×S permutation matrix via softsort, incurs a quadratic memory footprint $O(S^{2})$ with respect to the local neighborhood size *S*. This restricts its direct deployment on massive, dense outdoor LiDAR point clouds where neighborhood scales are exceptionally large. Future research will focus on alleviating this bottleneck by developing sparse permutation approximations and strictly linear-time scoring mechanisms. Furthermore, integrating our task-driven dynamic canonicalization directly into modern State Space Models (SSMs) to replace their predefined heuristics represents a highly promising avenue.

## Figures and Tables

**Table 1:** Summary of representative relevant works and their core characteristics.

Model (Year)	Core Mechanism	Advantages	Disadvantages
PointNet++ (2017) [10]	Hierarchical MLPs + Max-Pooling	Highly efficient baseline.	Context-agnostic before pooling.
DGCNN (2019) [12]	Dynamic Graph CNN	Captures local topology.	High *k*-NN computation cost.
ASSANet (2021) [17]	Anisotropic separable set abstractions.	Lower computational cost; improved accuracy.	Limited global context.
Point-BERT (2022) [20]	Masked Modeling + Transformer	Powerful pre-training.	Quadratic attention complexity.
PointNeXt (2022) [34]	Modernized PointNet++	Excellent speed-accuracy balance.	Still relies on context-agnostic pooling.
Point Transformer v3 (2024) [35]	Serialized Self-Attention	SOTA scaling for large scenes.	Serialization relies on fixed heuristics.
Point Cloud Mamba (2025) [27]	State Space Model (SSM)	Linear complexity; global context.	Relies on fixed space-filling curves.
Ours (SMA)	Learnable Sorting + MLP Mixing	Task-driven, context-aware, lightweight.	Slight overhead compared to pure MLPs.

**Table 2:** Classification results on ScanObjectNN (PB_T50_RS) and ModelNet40 datasets. Our SMA layer significantly boosts performance, especially on challenging real-world data, while maintaining model efficiency. ^†^ indicates pre-training. Bold numbers indicate the best performance. The upward arrow (↑) denotes the performance improvement compared to the baseline.

Method	ScanObjectNN (PB_T50_RS)		ModelNet40		Params	FLOPs
	OA (%)	mAcc (%)	OA (%)	mAcc (%)	(M)	(G)
PointNet [40]	68.2	63.4	89.2	86.2	3.5	0.9
PointNet++ [10]	77.9	75.4	91.9	-	1.5	1.7
PointNeXt-S [34]	87.7 ± 0.4	85.8 ± 0.6	93.2±0.1	90.8 ± 0.2	1.4	1.6
PointMLP [44]	85.4 ± 1.3	83.9 ± 1.5	**94.1**	**91.3**	13.2	31.3
PointMLP-Elite [44]	84.2	82.1	93.6	90.9	1.4	3.9
PointCNN [45]	78.5	75.1	92.2	88.1	0.6	-
DGCNN [12]	78.1	73.6	92.9	90.2	1.8	4.8
KPConv [14]	-	-	92.9	-	14.3	-
Point-BERT ^†^ [20]	83.1	-	93.2	-	22.1	4.8
Point-MAE ^†^ [21]	85.2	-	93.8	-	22.1	-
Point Transformer v3 [35]	86.3	83.9	-	-	-	-
PointVector [46]	87.8 ± 0.4	86.2 ± 0.5	-	-	1.55	-
Point Cloud Mamba [27]	88.1	**86.6**	93.4	90.7	34.2	45.0
SMA-tiny	86.0 ± 0.2	83.9 ± 0.3	92.9 ± 0.1	90.3 ± 0.4	**0.3**	**0.6**
PointNet++ (+SMA)	83.1 ± 0.7 (↑ 5.2)	84.2±0.8 (↑ 8.8)	92.8±0.3 (↑ 0.9)	89.9 ± 0.4	1.5	2.3
PointNeXt-S (+SMA)	**88.3** ± **0.3** (↑ 0.6)	86.5 ± 0.3 (↑ 0.6)	93.4 ± 0.1 (↑ 0.2)	90.9 ± 0.2 (↑ 0.1)	1.6	2.1
PointMLP-Elite (+SMA)	85.4 ± 0.2 (↑ 1.2)	83.0 ± 0.2 (↑ 0.9)	93.8 ± 0.1 (↑ 0.2)	91.1 ± 0.2 (↑ 0.2)	1.4	4.1

**Table 3:** Part segmentation results on ShapeNetPart. ^†^ indicates pre-training. Bold numbers indicate the best performance. The upward arrow (↑) denotes the performance improvement compared to the baseline.

Method	Ins. mIoU (%)	Cls. mIoU (%)
PointNet++ [10]	84.9	81.4
DGCNN [12]	85.2	82.3
Point-BERT ^†^ [20]	85.6	84.1
Point-MAE ^†^ [21]	86.1	84.2
PointMamba ^†^ [26]	86.2	84.4
PointNeXt-S [34]	86.4	84.4
PointMLP [44]	86.0	84.2
Stratifiedformer [47]	86.6	**85.1**
PointNet++ (+SMA)	85.5 (↑ 0.6)	82.6 (↑ 1.2)
PointNeXt-S (+SMA)	**86.7 (**↑ **0.3)**	84.6 (↑ 0.2)
PointMLP (+SMA)	86.4 (↑ 0.4)	84.5 (↑ 0.3)

**Table 4:** Semantic segmentation results on S3DIS Area-5. Bold numbers indicate the best performance. The upward arrow (↑) denotes the performance improvement compared to the baseline.

Method	mIoU (%)	OA (%)
PointNet++ [10]	53.5	82.8
DGCNN [12]	47.9	83.6
KPConv [14]	67.1	-
PointNeXt-S [34]	62.6	87.9
PointNeXt-B [34]	67.2	89.2
PointNeXt-L [34]	68.8	90.0
PointNet++ (+SMA)	54.5 (↑ 1.0)	83.2 (↑ 0.4)
PointNeXt-S (+SMA)	63.4 (↑ 0.8)	88.4 (↑ 0.8)
PointNeXt-B (+SMA)	67.5 (↑ 0.3)	89.6 (↑ 0.4)
PointNeXt-L (+SMA)	**69.1 (**↑ **0.3)**	**90.3** (↑ 0.3)

**Table 5:** Ablation on the serialization strategy. Bold numbers indicate the best performance. The learnable, anchor-based ordering demonstrates clear superiority over fixed, predefined heuristics.

Serialization Strategy	OA (%)
**SMA (Ours, Learned)**	**88.3**
Hilbert Curve [32] (Heuristic)	87.8
Z-order [33] (Heuristic)	87.3
No Sorting (Random)	85.7

**Table 6:** Ablation on the choice of the feature refinement gating function. Bold numbers indicate the best performance.

Gating Function	Output Range	OA (%)
**Sigmoid (Ours)**	[0,1]	**88.3**
2×Sigmoid	[0,2]	87.9
1+tanh	[0,2]	87.8
Identity (No Gate)	(−∞,∞)	87.5

## Data Availability

Data available in publicly accessible repository.

## Abbreviations

The following abbreviations are used in this manuscript:

MLP	Multi-Layer Perceptron
SA	Set Abstraction
OA	Overall Accuracy
mAcc	Mean Class Accuracy
mIoU	Mean Intersection over Union
